# A human, allogeneic cortical bone screw for distal interphalangeal joint (DIP) arthrodesis: a retrospective cohort study with at least 10 months follow-up

**DOI:** 10.1007/s00402-023-04785-2

**Published:** 2023-02-09

**Authors:** Christian Krasny, Christian Radda, Ralf Polke, Daniel Schallmayer, Gudrun H. Borchert, Christian Albrecht

**Affiliations:** 1 Orthopaedic Hospital Vienna-Speising, 1st Departement, Speisinger Straße 109, 1130 Vienna, Austria; 2 Dr. Borchert Medical Information Management, Egelsbacher Str. 39E, 63225 Langen, Germany

**Keywords:** Distal interphalangeal joint, DIP, Shark screw^®^, Human allograft cortical bone screw, Arthrodesis, Grip strength, Pinch strength

## Abstract

**Introduction:**

The prime requisites of a good digital arthrodesis are a painless and stable union in a proper position. Arthrodesis of the distal interphalangeal joint of the fingers is not without potential complications including nonunion, malunion, and deep tissue infections. The Shark Screw^®^ is a human, cortical bone allograft for osteosynthesis and an alternative to metal or bioabsorbable devices in orthopedics and trauma surgery. The primary hypothesis is that the fusion and complication rate, using the Shark Screw^®^, is at least similar to those reported in the literature, using metal or bioabsorbable screws.

**Material and methods:**

This retrospective cohort study analyzes the fusion and complication rate and the patient satisfaction of distal interphalangeal joint arthrodesis of 27 fingers with the human allogeneic cortical bone screw. Complications, Disabilities of Arm, Shoulder, and Hand Questionnaire (Quick-DASH) score and Michigan Hand Outcomes Questionnaire (MHQ) score, grip and pinch strength and fusion angle were investigated.

**Results:**

The mean follow-up was 23 months. At 6 weeks after surgery, fusion was obtained for all fingers. There was no surgical complication that required revision surgery. An average fusion angle of 13.6° ± 10.7° was measured. VAS pain score decreased significantly from 6.9 before surgery to 0.14 after surgery. The Quick-DASH score decreased from 10.7 to 7.8. The MHQ score improved in all sub-scores.

**Conclusion:**

The complication rates, using the Shark Screw^®^ for DIP joint arthrodesis, are lower compared to the results reported in the literature for other surgical techniques. Complications related to the human allograft cortical bone screw itself were not observed. The bone screw is completely remodeled into the host bone and further hardware removal is not necessary.

**Level of evidence:**

IV.

## Introduction

Distal interphalangeal (DIP) joint arthrodesis is usually performed to relieve pain, correct deformity, or stabilize a dysfunctional joint. Causes for such symptoms include acute traumatic or post-traumatic condition, osteoarthritis, and rheumatoid arthritis [1].

The Shark Screw^®^ is a human cortical bone allograft for the fixation of fractures, osteotomies, and arthrodesis. It is an alternative to metal or bioabsorbable devices in orthopedics and trauma surgery [2–5]. The safety of human allogeneic, sterilized bone transplants regarding disease transmission, biological tolerance, potential graft rejection, and allo-sensitization is well known, since allogeneic bone transplants (e.g., bone chips, bone blocks) are widely used in regenerative, maxillofacial and orthopedic surgery [6–9]. Besides the fixation function, due to its design as a set screw, the Shark Screw® exhibits osteoconductive properties promoting the ingrowth of blood vessels and bone cells [2, 8, 10]. The bone material, the human allogeneic cortical bone screw consists off, is completely remodeled into the patients bone. There is no need of hardware removal.

Flexion of the finger is important for the grip and pinch strength achieved after surgery [11]. A stable and pain-free DIPJ is necessary to guarantee the important functions of the finger.

There is insufficient evidence to support any particular technique [12, 13]. In most cases, successful fusion of the DIPJ improves the function and appearance of the digit with acceptable morbidity [13, 15]. Non-fusion-rate was 11% in patients with advanced osteoarthritis [16] and 15–0% in patients treated with K-wires [13]. DIPJ arthrodesis of the fingers is not without potential complications including nonunion, malunion, and deep tissue infections [18], chronic regional pain syndrome, and hypersensitivity. Stern et al. [19] evaluated the complications of different surgical techniques (crossed Kirschner pins, interfragmentary wire, longitudinal Kirschner pin and Herbert screw) and described 20% major complications and 16% minor complications [19].

The primary hypothesis of this retrospective study of DIPJ arthrodesis of 27 fingers in 21 patients is that the fusion rate, using the Shark Screw^®^, is at least similar to fusion rates reported in the literature achieved with metal or bioabsorbable screws. The secondary hypothesis is that the complication rate is not higher compared to other techniques. The advantage of complete bone integration without any hardware remnants will be shown.

## Patients and methods

### Patient data

For this study, a positive ethic vote was received (EK21-150-VK). 27 fingers in 21 patients with degenerative arthrosis were identified and included. Primary osteoarthrosis was the only indication for inclusion and other indications were exclusion criteria. The index finger was operated in 13 cases, the middle in 7, the ring finger in 3, and the small finger in 4 cases.

The primary outcome was the union rate, which was defined by the first author using common radiological criteria (signs of osseus union in the X-ray) and by a pain-free pinch grip 6 weeks after surgery. Fusion angles were measured on lateral radiographs. Age, profession, sex, dominant hand, operated hand, follow-up time, VAS pain score, Quick-DASH and MHQ score (pre-/post-surgery), grip and pinch strength for the operated and untreated hand, and patient satisfaction (1 = not satisfied, 2 = partly satisfied, 3 = satisfied, 4 = totally satisfied) were recorded. Grip strength and pinch strength were measured using the Jamar hand and finger dynamometer (Jamar, Hydraulic pinch gauge 52603, SEAHAN, Republic of Korea). In detail, to measure the crush grip strength, the hand dynamometer (Jamar, Hydraulic hand dynamometer SH5001) was used. For all subjects, the hand dynamometer was set at the hand hold level 2 out of 5. The patient had to sit in a stable position holding the forearm horizontal, the elbow flexed at 90°, and the wrist in a neutral position. First, the pinch grip strength was measured by squeezing the operated finger against the thumb with the finger dynamometer plates in between. The patients were instructed to apply their maximum strength for at least 3 s. The measured value of three repeating cycles of the finger with the fused DIP joint including the finger of the opposite hand was recorded. The patients had a 1 min break between each measurement. After a 5 min break, the crush grip strength was measured in the same way applying full power by clenching the hand into a fist while holding the hand dynamometer. Out of three repeated cycles, an average value was calculated for the operated and opposite fingers and hands.

### Surgical procedure

**Fig. 1:**
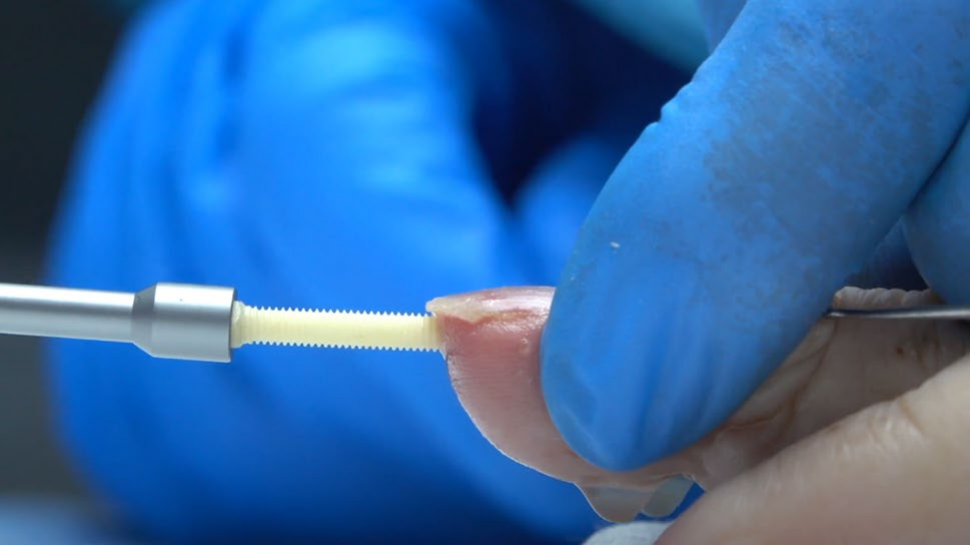
Arthrodesis of DIP with Shark Screw^®^; introducing the Shark Screw^®^

**Fig. 2:**
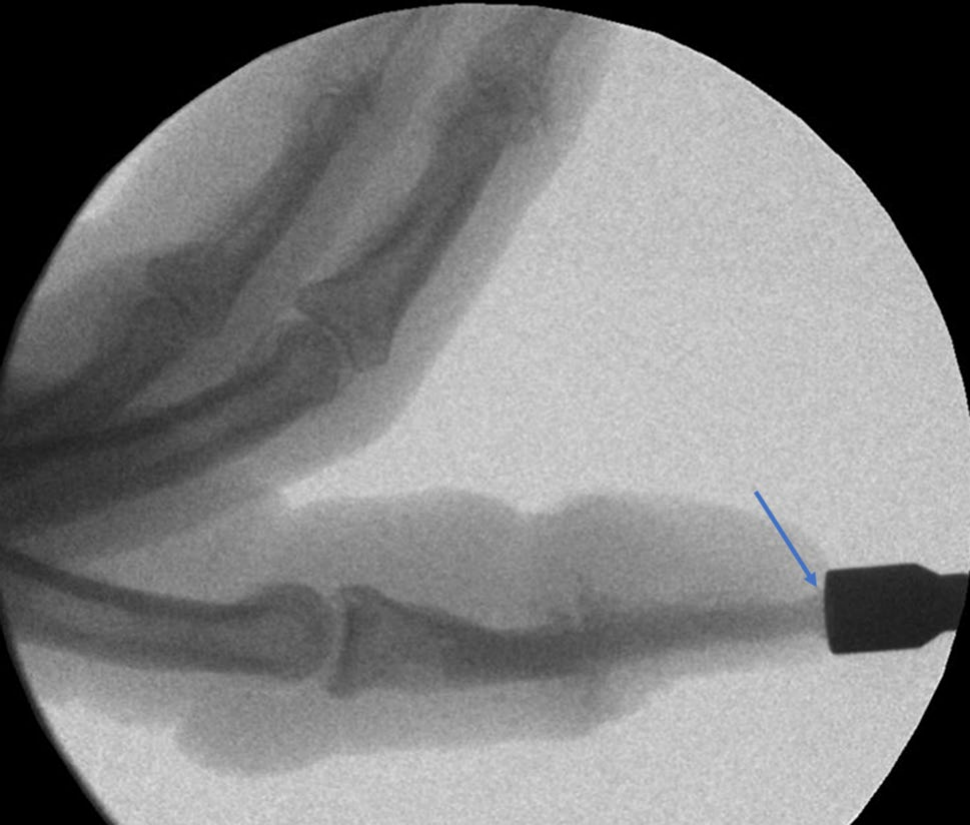
Insertion of the Shark Screw^®^, blue error shows the head of the Shark Screw^®^

**Fig. 3:**
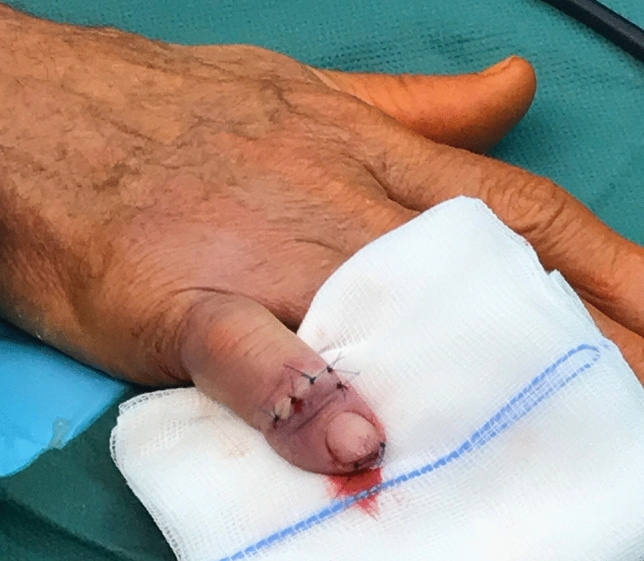
Skin closed with non-resorbable single stitch sutures

The surgical procedure was done under local anesthesia and using a finger tourniquet. An H-shaped skin incision on the dorsal side of the DIP joint was made. Subsequently, the tendon was cut longitudinally to expose the joint. Osteophytes were removed with a rongeur. Then any cartilage from the proximal and distal joint surfaces was removed and the 1.2 mm K-wire was used to achieve the desired fusion angle of the DIP joint under fluoroscopic guidance. Concerning the fusion angle, pinch grip finger function and cosmetic aspects were considered. After that, a small skin incision at the tip of the finger was performed, where a drill was introduced. Guided by the K-wire, the appropriate drill hole and the thread of a diameter of 3mm was done. Then the thread cutter and K-wire were removed. After lavage of the drill hole with NaCl solution, the Shark-Screw^®^ (3.5 mm diameter and 35 mm length, Surgebright GmbH, Lichtenberg, Austria) was introduced (Figs. 1 and 2). This screw dimensions were sufficient for all DIP joints in all patients treated with this technique. Joint position and placement of the screw were checked again with the image intensifier. After that, the head of the screw was shortened to bone level. Tendons were adapted again with two resorbable sutures and the skin was closed with non-resorbable single stitch sutures (Fig. 3). After surgery, a thin finger bandage and a custom-made finger sleeve was applied for 6 weeks.

### Postsurgical rehabilitation

The bandage was changed 2 weeks after surgery, and stitches removed. The same plastic finger sleeve was tightened up and used for the next 4 weeks. 6 weeks after surgery, an X-ray was taken, the finger sleeve was removed, and the patient was allowed to use his finger in daily life. The patient had three to five visits at our occupational therapists within the postoperative period of 6 weeks after removal of the stitches focusing on skin and scar care and mobilizing the neighbor joints.

### Statistics

Statistics were calculated with the program Origin Pro (OriginPro, Version 2022. OriginLab Corporation, Northampton, MA, USA). Because of non-normal distribution (analyzed with Kolmogorov–Smirnov test) for most of the data, Kruskal–Wallis–ANOVA was used for calculating statistically significant differences. Because there is only one study arm and all patients showed osseous union on the final X-ray, there was no power calculation to observe significant differences for unions. For VAS, Quick-DASH, and MHQ score before and after surgery, power was calculated and only when a power of > 0.8 was observed, significant differences were shown. Statistical significance was considered at a *p* value of < 0.05.

## Results

Patient demographics are described in Table 1.

**Table 1:** Patient data

Patient data	Total
Age [years]	64.6 ± 16.8 (22–87)
Operated site (right/left)	17/4
Right-handed/left-handed	17/4
Dominant hand involved (yes/no)	13/8
Work model	
Pension	11
Employed	8
Student	2
Male/female	4/17

Data are presented as mean ± SD (range)

Mean follow-up was 23 ± 9 months (Table 2). In two patients, minor complications were observed: one had a prolonged swelling and redness of the scar, and the other patient was cosmetically unsatisfied with the obtained fusion angle. A reduced fist clench with a fingertip hollow hand (FKHH) distance of more than 2 cm due to degenerative joint problems of the neighbor joints of the involved finger was recorded in two patients. Major complications like fractures of the implant or bone, pseudoarthrosis, superficial or deep infections, or skin problems were not observed in our cohort. Patient satisfaction was high (3.9, 4 = totally satisfied). All patients would repeat the surgery (Table 2).

**Table 2:** Clinical data and general data

Clinical data, general data		Data	
Follow-up [months]		23 ± 9	
Operated side (right/left)		17/4	
Range of fusion angle in three groups	0–10°	10–20°	> 20°
Observed average fusion angle within the three groups	4.9° ± 2.5°	12.1° ± 2.2°	29.3° ± 7.2°
Range of measured fusion angle in three groups	2–9°	10–17°	20–39°
Number of fused DIP joints assigned to the three groups of measured fusion angle	11	9	7
Number of observed complications* (yes/no)		4/17	
Patient satisfaction rating (1–4; 4 good))		3.9 (2.5–4)	
Would repeat this operation (yes/no)		21/0	

Data are presented as mean ± SD

*Complications were: fusion angle (1), hypoesthesia (1), FKHH 2 cm (2), redness, swelling (1)

**Fig. 4:**
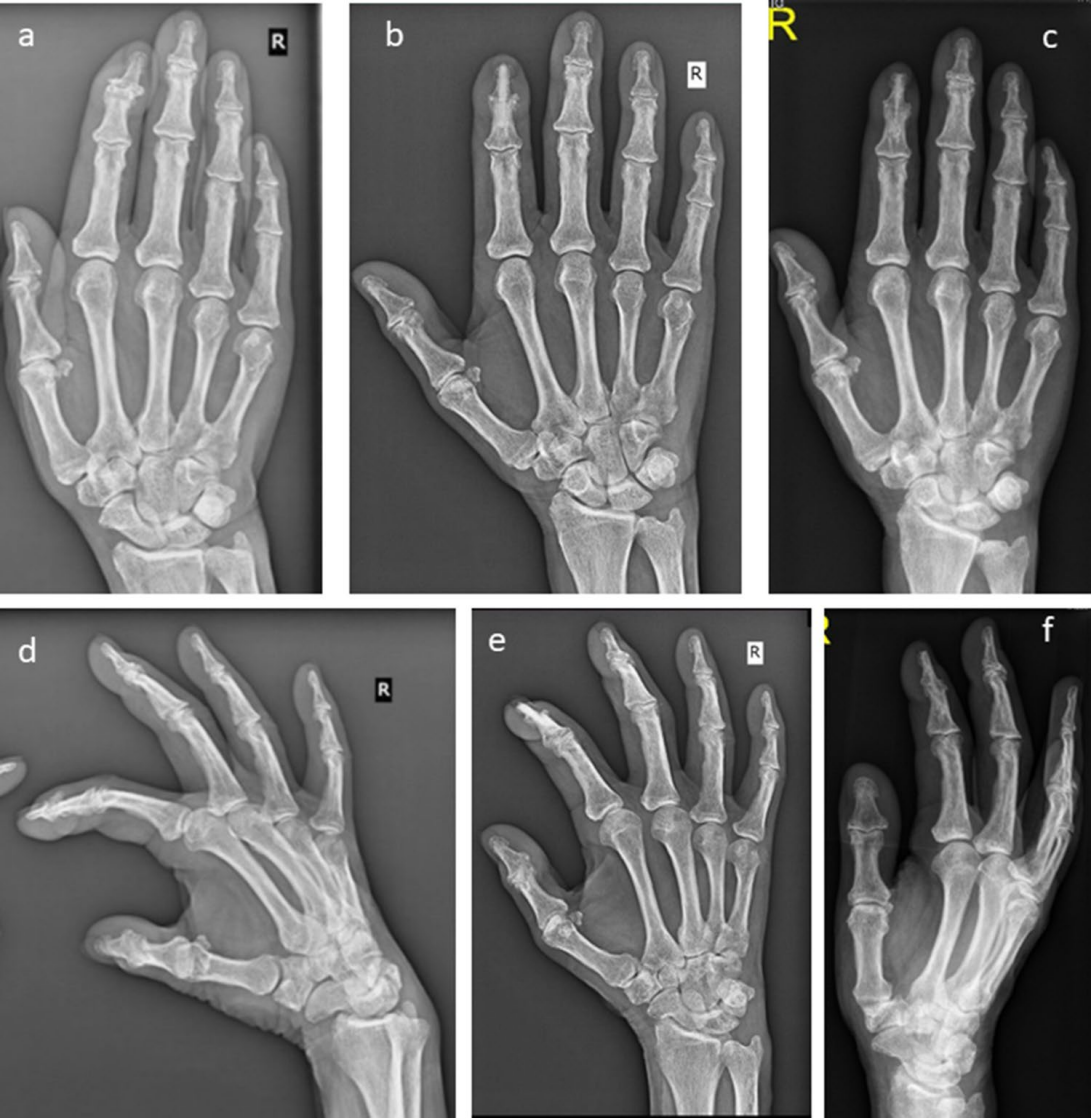
X-ray of DIP arthrodesis: **a**, **d**: pre-surgery; b, e: 6 weeks post-surgery; **c**, **f**: 1 year post-surgery, **a**, **b**, **c**: ap view, **d**, **e**, **f**: lateral view

Radiographs were performed before surgery (Fig. 4A), 6 weeks (Fig. 4B), and at least 6 months after surgery (Fig. 4C). Fluoroscopy was only performed during surgery.

Concerning the fusion angle, we informed the patients that a flexion angle between 10° (index finger) and 40° (little finger, [12, 13]) in the fused DIP joint is necessary to obtain the best pinch grip strength, but the final decision about the fusion angle was with the patient as described by other studies [13]. Factors like preoperative axis of the DIP joint, bone defects, or inaccuracy regarding the drilling and fixation with the screw implant led to deviations of the strived fusion angle. The obtained fusion angle was on average 13.6° ± 10.7°. VAS and MHQ values were statistically significantly improved after surgery (Table 3).

**Table 3:** Orthopedic scores

Score	Preoperative	Postoperative
VAS	6.9 ± 1.5	0.14 ± 0.48**
Quick-DASH	10.7 ± 14.5	7.8 ± 12.5
MHQ		
Function	81.4 ± 14.2	88.8 ± 13.1
Activities of daily living	83.1 ± 18.6	90.2 ± 15.2
Pain	75.5 ± 30.3	89.5 ± 18.8
Appearance (esthetic)	36.0 ± 32.7	82.7 ± 26.9**
Satisfaction	83.3 ± 19.4	93.2 ± 9.5
Total	76.3 ± 14.8	88.7 ± 12.4*

Data are presented as mean ± SD

Total MHQ: *p = 0.0035, 95% confidence interval (lower limit and higher limit), preoperative: 69.6 and 83.1, postoperative: 83.02 and 94.31

**p < 0.0001, 95% confidence interval (lower limit and higher limit), Vas Pain score: 6.2 and 7.6 pre-surgery and − 0.07 and 0.36 post-surgery

*MHQ* appearance: preoperative: 21,1 and 50,9, postoperative: 70.5 and 94.97

Grip strength of the operated hand and pinch grip strength of the operated finger were similar to the non-treated hand or fingers of the contralateral side (Table 4). Grip strength of male and female patients differed, but did not reach statistical significance.

**Table 4:** Clinical data (operated hand vs. non-treated hand

Clinical data	Operated hand	Non-treated hand
Grip strength [kg] all patients	29.2 ± 15.3	30.4 ± 14.8
Range	10–68	16–70
Male (*n* = 4)	44.0 ± 20.1	45.5 ± 16.8
Female (*n* = 17)	25.7 ± 12.2	26.8 ± 12.3
Pinch strength [kg]		
DIG II	3.9 ± 1.8, *n* = 13	4.5 ± 1.8, *n* = 13
Range	1.5–7.0	2.0–8.0
DIG III	1.6 ± 0.9, *n *= 7	1.8 ± 1.2, *n* = 7
Range	0.5–3.0	0.5–3.5
DIG IV	1.5 ± 0.5, *n* = 3	1.5 ± 0.9, *n* = 3
Range	1.0–2.0	0.5–2.0
DIG V	0.75 ± 0.5, *n* = 4	0.75 ± 0.5, *n* = 4
Range	0.5–1.5	0.5–1.5

Data are presented as mean ± SD

## Discussion

The most important finding of this study was that fusion was achieved in all 27 fingers of 21 patients after 6 weeks using the human, allogeneic cortical bone screw. In DIP arthrodesis, others report time to fusion between 8 and 11 weeks, using headless compression screws [15, 20, 21], reverse fix nails [22], and nitinol intramedullary fixation implants.[18]. Fusion was reported between 82 and 96% [12–18, 20, 22–26] for different fusion techniques. Stern et al. concluded that inadequate bone stock, inadequate bone resection, premature pin or screw removal, and infection had severe influence on bony healing and thus on the fusion [19]. They further state that no single technique has gained universal popularity for small-joint arthrodesis, and the arthrodesis technique did not determine whether union occurred [19]. The use of the human allogeneic cortical bone screws for arthrodesis can provide biological bridging of the arthrodesisgap, a stable fixation of the fused joint and must not be removed in case of septic complications [5]. Since the Shark Screw^®^ is a set screw with a thread pitch of 0.6 mm, it is absolutely rotation stable. The thread design complies with the biomechanical and physical laws of a set screw, where rotational stability is mandatory. In our study, all arthrodesis were fused after 6 weeks.

The mean follow-up was nearly 2 years and was described between 10 month and 3 years by others [22, 23, 25, 27].

There were no infections, bone healing problems, Shark Screw^®^ failure during or after surgery, and revision surgery in our patient cohort. Others report an infection rate of 4.5% [28] or 7% [15] and hardware failures and hardware removal [12].

In our study, the mean postoperative fusion angle of all DIP joints was 13.6° ± 10.7°. Auzias et al. published an average fusion angle measured on lateral X-rays of 9.3° (3°–19°) [17], which is similar to our findings. Furthermore, they described that in fingers with a 10° offset implant, the postoperative fusion angle was 10.7°(0–19°) and it was 5.6° (3°–11°) with straight implants [17]. In our study, except for one patient, all others obtained the planed and preferred fusion angle depending on functional and esthetic considerations (Table 2).

Grip strength of the dominant hand was for men 40–47 kg and for women 25–28 kg depending on the age group [29, 30]. Grip strength of the operated hand in our study was on average 29 kg and was similar to that in other reports.[29–31]. Gugger et al. [32] reported a grip strength of 21.2 kg post-surgery, which equals 69% of the untreated side. Liu et al. reported also a lower grip strength, 26.4 kg, post-surgery [33]. A grip strength of 91–96% of the untreated side is supported by others [11, 31]. In our patient cohort, the grip strength was 96% of the untreated side and not related to the fusion angle. Song et al. described a pinch power of 75% of the untreated side [21], while we obtained a pinch power between 87 and 100% of the untreated side. A pinch strength of 5.5 kg was reported for the index finger by Liu et al. [33] and was higher than the one we recorded for the untreated hand (4.5 kg).

There is a potential risk to penetrate or burst the cortical bone of the distal phalanx with a screw of a bigger diameter than the phalanx, especially in little or very small fingers. Due to the fact that the Shark Screw^®^ consists of human cortical bone, small bone defects or cracks of the cortical bone will be bridged with the screw itself and biological healing would not be disturbed. Extensive shortening of the finger, as described for other techniques [13], can be prevented in cases of reduced bone contact in the arthrodesis gap, because the cortical bone screw ensures a stable structure which supports further bone healing to obtain a solid arthrodesis. These might be unique advantages of the human, allogeneic cortical bone screw and could reduce the risk of incomplete bone healing in such cases.

Our patient cohort was older than described in most other studies [17, 18, 22–27], and as reported more female than male patients were treated [24, 27]. The pre- and postoperative VAS and MHQ improved as described elsewhere [16–18, 23, 25, 27, 33]. Additionally, there was a trend to better Quick-DASH post-surgery. Patient satisfaction is generally high after this procedure and was 3.9 in our analyses and 4 ± 1 in the study of Trumble et al. [34].

No attention to allergies, such as nickel [25], has to be given, when using the human, allogeneic cortical bone screw. This avoids a particle-induced aseptic implant loosening [25]. No inflammatory infiltrates were observed 10 weeks after implantation of the cortical bone screw for treating a hallux rigidus [10].

## Strength of the study

The strength of the study is that no pseudarthrosis was recorded in all evaluated patients, regardless of the finger treated and the indication of the surgery. Cases with insufficient bone stock due to defects or incomplete adaptation of the arthrodesis gap seem to benefit from the biological properties of the screw itself concerning bony consolidation. In the interest of the patient, clinical practice should change to this new technique, resulting in normal bone after arthrodesis.

## Limitations of the study

Limitations of the study such as a small number of patients and a single surgeon treatment are evident. Another limitation is that there is just one study arm and no control group.

In conclusion, the human, allogeneic cortical bone screw (Shark Screw^®^) offers an option for DIPJ arthrodesis. Complication rates seems to be lower, compared to conventional metallic or biodegradable magnesium implants because implant-associated side effects could be completely avoided. 6 weeks post-surgery, all patients of our series had complete fusion of the DIP joint. The bone screw transforms into original bone starting at the moment of implantation and is completely remodeled within 6–12 months. A unique advantage of the Shark Screw^®^ seems to be the fact that bone defects of the cortical bone or reduced adaptation of the fusion site can be bridged with the screw. They heal stably without consequences by transforming into the host bone structure. The introduced bone screw works like a biological guiding structure and supports the bone healing by itself. Further investigations concerning the defect size, which can be bridged with the Shark Screw^®^, to get stable biological bone healing, have to be done. Another benefit is that the bone healing itself is not affected by the implant, and thus no complications are expected related to the implant und further hardware removal is avoided. All these advantages could potentially decrease the cost for the social security system.

## Data Availability

All data recorded are presented in the manuscript. Additional information can be obtained from the corresponding author on request.
